# A Rheological Study of the High-Temperature Properties of Fast-Melting SBS/Epoxy-Modified Asphalt Binders

**DOI:** 10.3390/polym17050581

**Published:** 2025-02-22

**Authors:** Lei Feng, Xinyong Zhang, Tianyu Sha, Decai Wang, Ben Niu, Riran Wang, Xiyang Hou

**Affiliations:** 1 Taihang Urban and Rural Construction Group Co., Ltd., 569 Shitong Rd., Shijiazhuang 050200, China; thcxnb@126.com (L.F.); yangl20230802@126.com (X.Z.); weijw0820@126.com (B.N.); 2 School of Water Conservancy and Transportation, Zhengzhou University, 100 Science Rd., Zhengzhou 450001, China; shaty007@163.com (T.S.); wangrr@zzu.edu.cn (R.W.); 3 School of Civil Engineering and Communication, North China University of Water Resources and Electric Power, Zhengzhou 450045, China; houxiyang03@163.com

**Keywords:** novel fast-melting modifier, asphalt binder, high-temperature performance, epoxy resin, rheological model

## Abstract

A fast-melting epoxy resin styrene–butadiene–styrene composite modifier (ER-SBS-T) was utilized for the rapid modification of an asphalt binder. The effect of this novel fast-melting modifier on high-temperature performance is not supported by any pertinent investigations. First, the penetration, softening points, and 60 °C kinematic viscosity of the asphalt samples were tested. In addition, these asphalt samples were subjected to multiple stress creep recovery (MSCR) tests to assess the ability to recover from creep and to test the high-temperature performance of the asphalt binder. The study then compared different models based on the zero-shear viscosity (ZSV) test. The research shows that the use of ER-SBS-T composite modifiers significantly improves the high-temperature performance of asphalt binder. The high-temperature performance of fast-melting SBS-T-modified asphalt binder is comparable to that of SBS-modified asphalt binder. The high-temperature performance of the asphalt binder can still be significantly improved when the amount of ER-SBS-T composite modifier exceeds 6%. For the high-temperature performance grading of the ER-SBS-T composite-modified asphalt binder, a reference temperature of 70 °C can be established. The results also demonstrate that the Cross rheological model is more suitable for determining the zero-shear viscosity of the ER-SBS-T composite-modified asphalt binder.

## 1. Introduction

As one of the most common asphalt pavement diseases, rutting has become the focus of asphalt pavement disease management. Asphalt binder plays an important role in the rutting resistance properties of asphalt mixtures [1]. Accordingly, research into the high-temperature performance of asphalt binder has also become a long-term topic of pavement research. The modifier material is one of the most important factors influencing the high-temperature performance of asphalt binder [2]. As the most widely used modifier, SBS modifier can meet the high-temperature performance requirements of conventional pavements [3]. However, the SBS-modified asphalt mix produced by the conventional process should have styrene–butadiene–styrene (SBS) modifiers added to the asphalt binder in advance. The asphalt mix behaves poorly during storage and transport. It is prone to segregation and ageing, which significantly reduces the performance of the asphalt mix [4]. Epoxy asphalt binder is a modified asphalt binder that meets the high-temperature performance and thermosetting properties of heavy-traffic pavements. It has been demonstrated that epoxy asphalt binder generally exhibits superior high-temperature performance compared to various other modified asphalt binders [5]. Epoxy asphalt binders typically consist of epoxy resin, curing agent, and asphalt binder. However, epoxy asphalt binder increases the difficulty in the actual construction process due to excessive viscosity, and the curing reaction of epoxy asphalt needs to be carried out at a high temperature, which requires a lot of energy [6,7].

To overcome problems in the conventional construction process of asphalt mixtures, to prevent deterioration of properties during transport, and to maintain the high-temperature performance of the asphalt mixtures, some researchers have developed an asphalt modifier (dry method) that can be added to the mixing tank so that it can be mixed directly into the asphalt mixtures. Luo et al. [8] provided a technique for the rapid expansion of SBS-modified asphalt binder in the dry modification mode. This technique improves the stability of SBS-modified asphalt. Some researchers have added a rapid-melting component (chemical reagent) to the SBS modifier to prepare a granular SBS-T asphalt modifier that can achieve rapid melting. Some researchers have combined SBS-T with epoxy resin to meet the needs of heavy traffic pavements. SBS-T and epoxy resin make a cross-linking reaction with various types of curing agents at high temperatures. They form a composite polymer with a three-way lattice structure, known as the fast-melting epoxy–styrene–butadiene–styrene (ER-SBS-T) modifier.

There has been some research into the high-temperature performance of modifiers using the dry method. Yin et al. [9] investigated the high-temperature stability of polyolefin/ethylene copolymer-modified hot mix asphalt (dry method). The test results show that the dynamic stability improved compared to the SBS asphalt mix. Li et al. [10] prepared an epoxy asphalt modifier (dry method) and tested the high-temperature performance of the epoxy asphalt mix (dry method). The results confirmed that the epoxy asphalt (dry method) mix has good high-temperature performance.

Improving the rheological properties of asphalt binder is considered a key strategy to reduce rutting [11]. Existing research into the high-temperature performance of modifiers (dry method) has mainly focused on the macro indexes of asphalt binder and asphalt mixes, which reflect only part of the high-temperature properties of asphalt binder. This cannot fully reflect the role of the asphalt binder in the asphalt pavement. Existing research lacks methods to adequately evaluate the high-temperature behaviour of such modified asphalts (dry method). The existing rheological method is mainly based on a dynamic shear rheometer (DSR) [12]. The rutting parameter in the Strategic Highway Research Programme (SHRP) has often been used to evaluate the high-temperature performance of modified asphalt binder. The elastic recovery of asphalt has not been fully considered in the evaluation of high-temperature performance [2,13,14]. The rutting parameter is obtained from the DSR time scan test at a given temperature and is also used in the Superpave performance grading (PG) system. The PG grade selected for a heavy traffic pavement is usually one grade higher than that for a standard traffic pavement. Some researchers [15,16] have concluded that the temperature of the pavement has not reached the corresponding grading temperature, so this performance grading cannot reflect the high-temperature properties of asphalt in heavy traffic pavements.

To overcome the limitations of the rutting parameter in evaluating the high-temperature performance of asphalt binder, some new parameters [17,18,19,20] have been proposed in recent years to evaluate the rheological properties of asphalt, such as the zero-shear viscosity (ZSV), low-shear viscosity (LSV) and non-recoverable creep compliance. Among these parameters, researchers [21,22,23,24] indicate that the non-recoverable creep compliance obtained from the MSCR test is better related to rutting. The parameters based on MSCR can reflect the rutting very well. The zero-shear viscosity (ZSV) test is widely used. This is because it can give a good indication of the high-temperature performance of asphalt binder [25]. In addition, some research has shown that the pavement performance classification based on the MSCR test can more clearly reflect the performance of heavy duty and heavy traffic pavements [26].

No investigations have yet been carried out on the rheological properties of SBS-T-modified asphalt binder and epoxy resin–SBS-T composite-modified asphalt binder. This research included a penetration test, a softening point test, a zero-shear viscosity (ZSV) test based on a dynamic shear rheometer (DSR), and a multiple stress creep recovery (MSCR) test based on DSR equipment. Comparative studies need to be carried out on ER-SBS-T composite-modified asphalt binder, SBS-T-modified asphalt binder, SBS-modified asphalt binder and 70# base asphalt binder. This research selected more appropriate parameters to evaluate high-temperature properties. In addition, the effect of modifier loading and temperature on the high-temperature performance of the ER-SBS-T composite-modified asphalt binder was investigated. A more appropriate zero-shear viscosity model was chosen for this study. The reference temperature for the pavement performance classification of the ER-SBS-T composite-modified asphalt was obtained. The pavement performance grading of ER-SBS-T composite-modified asphalt was split. This study will provide a technical guide to the high-temperature performance of ER-SBS-T composite-modified asphalt binder.

## 2. Materials and Methods

### 2.1. Materials

**Figure 1:**
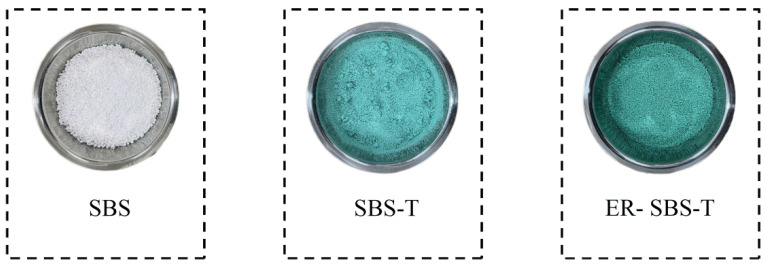
Physical image of the three types of modifiers.

The base asphalt binder (70#) was used in this investigation, and its technical information is shown in Table 1. The ER-SBS-T composite modifier, SBS-T modifier, and SBS modifier selected in this investigation were all supplied by Guolu Gaoke (Beijing, China) Engineering Institute Co., Ltd., and the technical information is given in Table 2. The physical representation of the three types of modifiers is illustrated in Figure 1.

### 2.2. Sample Preparation

The mass mixing ratio of each modifier (including SBS, SBS-T, ER-SBS-T) relative to the base asphalt binder was 4%, 6%, 8%, 10%, 12%. The preparation process for the three modified asphalt binders was as follows. First, a constant temperature of 165 °C was maintained for the 70# base asphalt binder. The SBS modifier, the SBS-T modifier and the ER-SBS-T complex modifier were then weighed separately at different dosages. After the addition of the modifiers to the base asphalt binder, the mixture was sheared for 30 min at a speed of 4500 rpm, while maintaining an asphalt temperature of 160 ± 10 °C. Finally, the prepared modified asphalt was cured at 160 °C for 60 min.

### 2.3. Penetration and Softening Point Test

The modifiers SBS, SBS-T, and ER-SBS-T were selected for this study and were added at 4%, 6%, 8%, 10%, and 12%. The blending ratio here refers to the mass ratio of modifiers to asphalt binder. The softening point test refers to T0606-2011, and the penetration test refers to T0604-2011.

### 2.4. 60 °C Kinematic Viscosity Test

In China, the temperature of asphalt pavement in summer can often reach more than 60 °C, so the 60 °C kinematic viscosity can characterize the rutting resistance of asphalt mixture, which is a very important high-temperature performance index. The 60 °C dynamic viscosity test is a commonly used experimental method to test the viscosity of asphalt at 60 °C using a dynamic viscosity tester. The specific test procedure is carried out according to the method of T0620-2000 in the Test Procedure of Highway Engineering Asphalt and asphalt mixture JTGE20-2011 [27].

### 2.5. Multiple Stress Creep Recovery (MSCR) Tests

**Figure 2:**
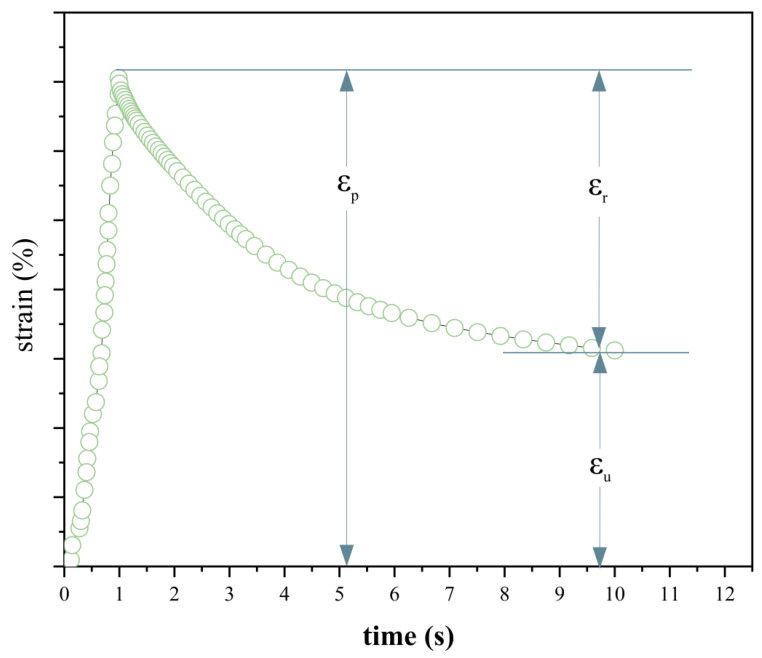
Schematic diagram of the MSCR loading test.

The DHR-1 dynamic shear rheometer supplied by TA was used for the multiple stress creep recovery (MSCR) tests. The samples in this test are the ER-SBS-T composite modifier, SBS-T modifier, and SBS modifier. The temperature was set at 58 °C, 64 °C, and 76 °C, with a temperature interval of 6 °C. The 25 mm fixture was selected. The distance between the two fixtures was 1 mm. It was loaded for 1 s and unloaded for 9 s at 0.1 kPa and 3.2 kPa. This was repeated for 20 cycles at 0.1 kPa. It was repeated for 10 cycles at the shear stress level of 3.2 kPa. The MSCR test has 6 parameters. For the 0.1 kPa stress level, there was an average percent recovery of R_0.1_, the average non-recoverable creep compliance J_nr0.1_. For the 3.2 kPa stress level, the average percent recovery was R_3.2_ and the average non-recoverable creep compliance was J_nr3.2_. The percentage difference in non-recoverable creep compliance was J_nr-diff_. The percentage difference in average percent recovery was R_diff_. A schematic representation of the MSCR of an asphalt binder sample during a loading cycle is shown in Figure 2. The formula is as follows.

(1) $$R=\frac{\varepsilon_{p}-\varepsilon_{nr}}{\varepsilon_{p}-\varepsilon_{0}}$$

(2) $$J_{nr}=\frac{\varepsilon_{nr}-\varepsilon_{0}}{\tau }$$

(3) $$R_{diff}=\frac{R_{0.1}-R_{3.2}}{R_{0.1}}\times 100\%$$

(4) $$J_{nr-diff}=\frac{J_{nr3.2}-J_{nr0.1}}{J_{nr0.1}}\times 100\%$$

where $\varepsilon_{nr}$ is residual strain, $\varepsilon_{0}$ is initial strain, $\varepsilon_{p}$ is peak strain, τ is loading stress.

To visually and effectively analyze the influence of various factors on the MSCR indexes, SBS-modified asphalt binder with 6% modifier content, SBS-T-modified asphalt binder with 6% modifier content, and ER-SBS-T composite-modified asphalt binder with 4%, 6%, 8%, 10%, and 12% modifier content were selected as samples. To investigate the effectiveness of modifier content, temperature, and other factors on the high-temperature performance of the modified asphalt binder, three different temperatures, i.e., 58 °C, 64 °C, and 70 °C, were selected to more accurately imitate the temperature of the actual pavement.

### 2.6. Zero-Shear Viscosity (ZSV) Test

**Figure 3:**
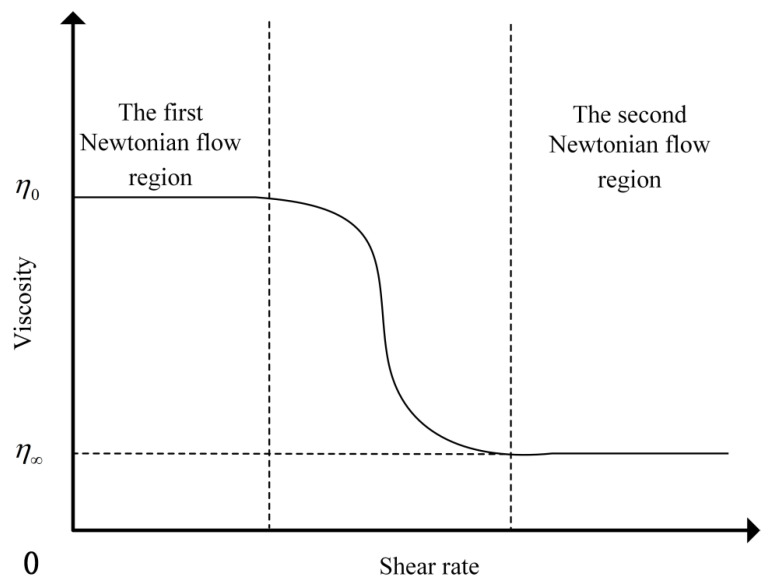
Schematic diagram of viscosity at different shear rates.

Zero-shear viscosity is the first index proposed by European countries to evaluate the high-temperature performance of asphalt binder. Zero-shear viscosity refers to the viscosity value of pitch at the zero-shear rate. During the test, the asphalt binder exhibits the characteristics of a non-Newtonian fluid in which the viscosity gradually increases as the shear rate decreases. The viscosity versus shear rate curves of non-Newtonian fluids are divided into the first Newtonian flow region and the second Newtonian flow region. When the shear rate decreases and the rising viscosity curve becomes stable, the viscosity is zero-shear viscosity [28]. The viscosity of the sample at different shear rates is shown in Figure 3.

There are many methods to determine the zero-shear viscosity of asphalt, including the static load test method and the dynamic load test [29,30]. In this study, the frequency scanning method was adopted in the dynamic load test. The load frequency was 0.1–100 Hz, the strain level was 5%, and the test temperature was 60 °C, and the Cross and Carreau models were used to predict the ZSV values of asphalt binders. The mathematical expressions of the Cross and Carreau models are given in Equations (5) and (6). The sample was an ER-SBS-T composite-modified asphalt binder with modifier content of 4%, 6%, 8%, 10%, and 12%, SBS-modified asphalt binder with modifier content of 6%, and SBS-T-modified asphalt binder with modifier content of 6%.

(5) $$\frac{\eta -\eta_{\infty }}{\eta_{0}-\eta_{\infty }}=\frac{1}{1+{\left(k\omega \right)}^{m}}$$

(6) $$\frac{\eta -\eta_{\infty }}{\eta_{0}-\eta_{\infty }}=\frac{1}{{\left[1+{\left(k\omega \right)}^{2}\right]}^{\frac{m}{2}}}$$

where η = asphalt viscosity, Pa·s.; $\eta_{0}$ = zero-shear viscosity, Pa·s; $\eta_{\infty }$ = the second Newton viscosity, Pa·s; K = material characteristic parameter; m = material characteristic parameter; ω = shear rate, 1/s.

## 3. Results and Discussion

### 3.1. Test Results for Penetration and Softening Point

**Figure 4:**
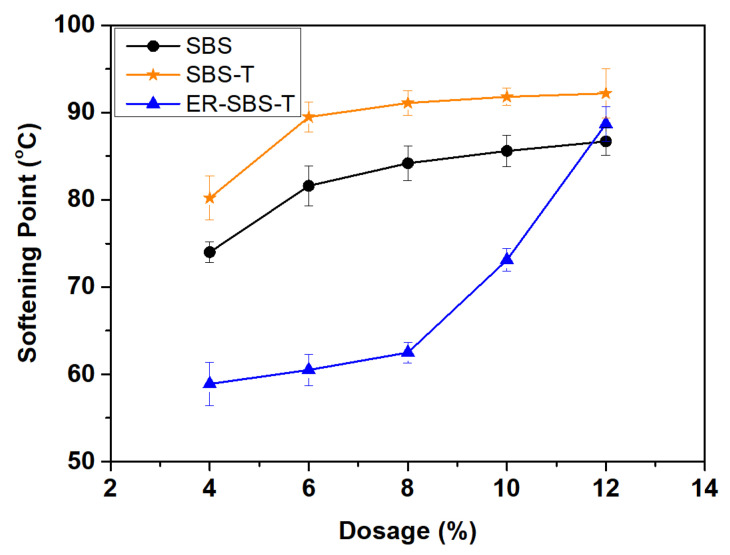
The relationship between the proportion of modifier blending and softening point.

The asphalt softening point test uses the ball and ring method to test the softening points of the modified asphalt to evaluate the high-temperature performance of the modified asphalt. To more intuitively reflect the influence trend of the SBS modifier, SBS-T modifier, and ER-SBS-T composite modifier on the softening points of modified asphalt and compare the softening points of different types of modified asphalt, the test results are presented in Figure 4.

The softening point of the 70# base asphalt is 47.7 °C, as illustrated in Figure 4. After the addition of these three modifiers, the softening point is improved, indicating that these three modifiers can improve the high-temperature performance of asphalt binder, as stated in some studies [31]. With the increase in the dosage of modifiers, the softening point of the three types of modified asphalt binder gradually increased.

When the dosage of SBS modifier and SBS-T modifier is between 4% and 6%, the softening point of the prepared modified asphalt binder increases rapidly. When the dosage of SBS modifier and SBS-T modifier is more than 6%, the rate of improvement of the softening point of the prepared modified asphalt slows down, indicating that the improvement effect of the modifier on the asphalt softening point is not great at this time [32]. When the dosage of ER-SBS-T composite modifier is between 4% and 8%, the softening point of the prepared modified asphalt is slow to increase, which may be because the epoxy resin is difficult to fully react to form a three-way lattice structure at the lower dosage, and the asphalt phase cannot form a more perfect compatible system with the epoxy phase [33,34]. It has been demonstrated that the epoxy resin gradually commences to exert the modification mechanism once the amount of ER-SBS-T composite modifier exceeds 8%. A new equilibrium system is formed between asphalt and epoxy resin and SBS-T modifiers, resulting in a sudden increase in the softening point.

By comparing the effect of the SBS modifier with the SBS-T modifier on the asphalt softening point. As can be seen, both modifiers have excellent improvements on the asphalt softening point and the change trend is similar. Both at a dosage between 4% and 6%, the asphalt softening point was promoted rapidly. After 6% the rate of increase slows down, suggesting that the composition of the two modifiers is similar. The mechanism of action to improve the softening point is similar. However, in comparison, the degree of improvement in the asphalt softening point of the SBS-T modifier at different dosages is slightly higher than that of the SBS modifier at the same dosage. This may be because the fast-melting component in the SBS-T modifier helps to improve the compatibility of the asphalt, according to some research [14].

Comparing the influence of the three modifiers on the asphalt softening point, it can be seen that the degree of improvement of the ER-SBS-T composite-modified asphalt binder at low dosage is lower than that of the other two asphalt binders. As it is difficult for the epoxy resin to fully react to form the three-dimensional network structure, the modification mechanism cannot be fully developed. After the dosage of modifiers is more than 10%, the softening point of ER-SBS-T composite-modified asphalt binder gradually exceeds that of SBS-modified asphalt binder and has a tendency to exceed that of the SBS-T-modified asphalt binder. The softening point of the two SBS modified asphalt binders is not significantly improved when the modifier dosage is 6%, and the softening point of the ER-SBS-T composite-modified asphalt binder is still significantly improved when the modifier dosage is 12%. This indicates that the high dosage of ER-SBS-T composite modifier can still fully blend into the asphalt and significantly improve the softening point of the asphalt. Therefore, the high dosage ER-SBS-T composite modifier has better high-temperature performance than the SBS modifier and can adapt to a more extreme high-temperature pavement environment.

**Figure 5:**
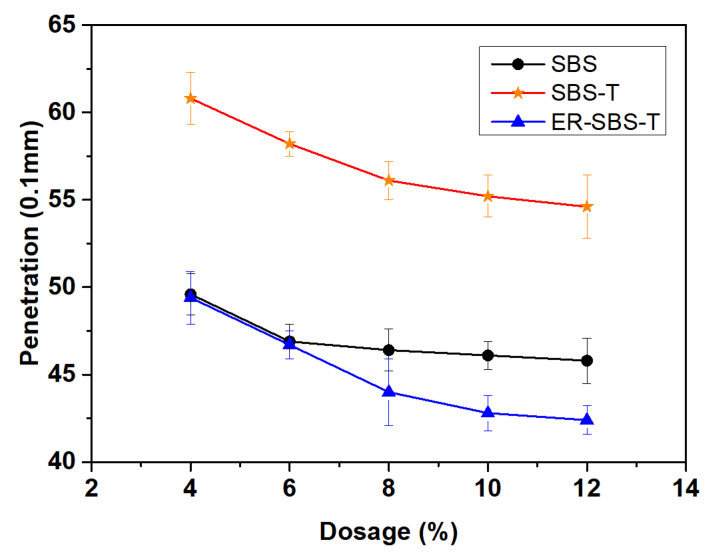
The relationship between the proportion of modifier blending and penetration.

The temperature of the asphalt penetration test is 25 °C. The penetration of various modified asphalt binders was tested. To directly reflect the influence of the SBS modifier, SBS-T modifier, and ER-SBS-T composite modifier on the permeability of the modified asphalt binder and to compare the penetration of different modified asphalts, the results are given in Figure 5.

The penetration of the 70# base asphalt is indicated in Table 1 to be 72.4 (0.1 mm), while the penetration of the three modified asphalt binders is lower than that of the 70# asphalt binder. This suggests that the three modifiers can increase the consistency of the asphalt at room temperature. As the dosage increased, the penetration of the three modified asphalt binders gradually decreased.

By observing the changing trend of the penetration of these three modified asphalt binders, it was found that the dosage is between 4 and 12%, these three types of modified asphalt binder penetration decrease are not more than 1 mm, penetration change trend is slow. It shows that the dosage of the three types of modifiers is high. The 25 °C penetration test cannot significantly reflect the high-temperature performance of the modified asphalt binder.

Comparing the effects of the three modifiers on asphalt penetration, it can be seen that the ER-SBS-T composite modifier has the best effect on asphalt penetration, the SBS modifier has the second-best effect and the SBS-T modifier has the worst effect, and the difference in penetration between the SBS-T-modified asphalt binder and the other two modified asphalt binders is obvious. This may be related to the material characteristics of the SBS-T modifier. This is because the rapid melting component in the SBS-T modifier cannot fully function at normal temperatures, making the modified asphalt soft at normal temperatures. At high temperatures, however, the rapid melting component promotes the phase fusion of SBS and asphalt. The SBS-T-modified asphalt binder therefore has a high softening point. It is concluded that it is not appropriate to evaluate the high-temperature performance of SBS-T-modified asphalt binder using a 25 °C penetration test.

### 3.2. 60 ° C Kinematic Viscosity Outcomes

**Figure 6:**
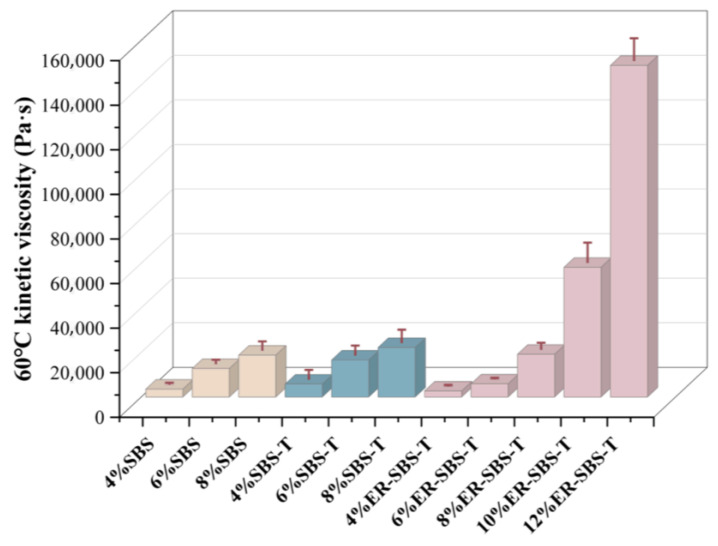
60 °C kinematic viscosity changes in the modified asphalt binder.

The relationship between viscosity and rheological properties of fluids is studied, as these two indicators can characterize the ability of fluids to resist flow deformation. In China, the temperature of asphalt pavements in summer can often reach 60 °C or higher, so 60 °C kinematic viscosity can characterize the high-temperature performance of asphalt. The 60 °C kinematic viscosity test is a common experimental method used to test the viscosity of asphalt. The 60 °C kinematic viscosity test can reflect the rutting resistance of the asphalt mix, and the 60 °C kinematic viscosity is a very important high-temperature performance indicator. The viscosity of the high-content SBS and the SBS-T-modified asphalt binder is relatively high at 60 °C, which results in a longer test time and lower shear rate compared to a lower content, resulting in a greater error in the 60 °C kinematic viscosity test results. And the penetration and softening point indices of SBS-modified asphalt binder and SBS-T-modified asphalt binder show a significant decrease in their rate of variation when the modifier content exceeds 6%. Furthermore, these indices remain unchanged when the modifier content exceeds 8%. Therefore, in this experiment, the SBS-modified asphalt binder and SBS-T-modified asphalt binder with modifier contents of 4%, 6%, and 8% and ER-SBS-T composite-modified asphalt binder with modifier contents of 4%, 6%, 8%, 10%, and 12% were tested. The measured 60 °C kinematic viscosities and their variation rates are displayed in Table 3. To provide a clearer comparison of the 60 °C kinematic viscosity changes in the different modified asphalts, the data from the table were plotted in a bar graph as shown in Figure 6.

Table 3 summarizes these conclusions: as the dosage of the three modifiers increases, the 60 °C kinematic viscosity of the three modified asphalt binders gradually increases, but the rate of change varies for the different modified asphalt binders. The rate of change for SBS-modified asphalt reaches its maximum at a dosage of 6%, which is 258%. The rate of change for SBS-T-modified asphalt binder is the highest at 6%, which is 271%. The rate of change for the ER-SBS-T composite-modified asphalt binder is highest at 10%, which is 441.3%. Both SBS and SBS-T modifiers show a significant increase in high-temperature performance at a dosage of 6%, indicating that the modification mechanism of the SBS and SBS-T modifiers is similar, both forming a more complete structure at 6%. However, the 60 °C kinematic viscosity of the ER-SBS-T composite-modified asphalt binder rises sharply at a dosage of 10%, because at 10% the materials, such as epoxy resin and the curing agent, undergo a curing reaction, significantly increasing the kinematic viscosity of the asphalt binder. The rate of change for the SBS- and SBS-T-modified asphalt binder decreases significantly at a dosage of 8%, indicating that the addition of these SBS and SBS-T modifiers is no longer significant at this point. Considering the actual cost of construction and other factors, researchers often choose a dosage of 6% to study the high-temperature performance of SBS-modified asphalt.

Figure 6 indicates that the 60 °C kinematic viscosity of SBS-T-modified asphalt binder is higher than that of SBS-modified asphalt binder at the same loading rate. This indicates that although SBS-T and SBS have similar modification mechanisms, the unique rapid melting property of SBS-T helps to increase the 60 °C kinematic viscosity of the modified asphalt. However, when the ER-SBS-T composite-modified asphalt binder was blended at 4% and 6%, the 60 °C kinematic viscosity was lower than that of SBS- and SBS-T-modified asphalt binder at the same dosage, which is consistent with the results of the softening point test, confirming that the high-temperature performance of low-dosage ER-SBS-T composite-modified asphalt binder is poor. Nevertheless, the 60 °C kinematic viscosity of the ER-SBS-T composite-modified asphalt binder at 10% loading is 58,000 Pa∙s, while that of the SBS-modified asphalt binder at 8% loading is only 18,900 Pa∙s. Furthermore, the 60 °C kinematic viscosity of ER-SBS-T composite-modified asphalt binder at 12% loading is 148,900 Pa∙s, which is much higher than that of 8% SBS-T-modified asphalt binder. Therefore, the high-temperature performance of ER-SBS-T composite-modified asphalt binder at high loading is more valuable to study. However, due to the difference in the type and dosage of modifiers, there is a difference in the instrument shear rate. Therefore, it is still debatable whether the 60 °C kinematic dynamic viscosity data obtained are accurate and whether cross-comparison studies of modified asphalt can be carried out.

### 3.3. Temperature and Stress Level on MSCR-Related Parameters

**Figure 7:**
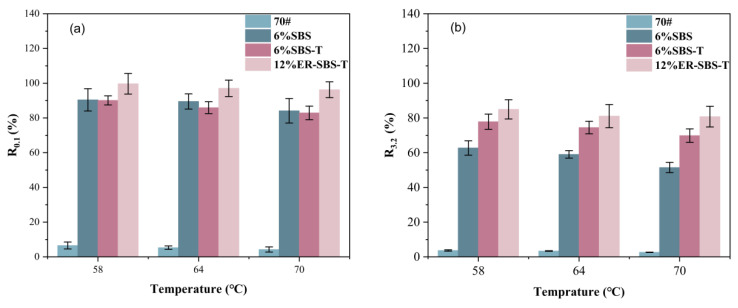
The average percentage recovery under different stress levels and different temperatures. (**a**) 0.1 kPa, (**b**) 3.2 kPa.

**Figure 8:**
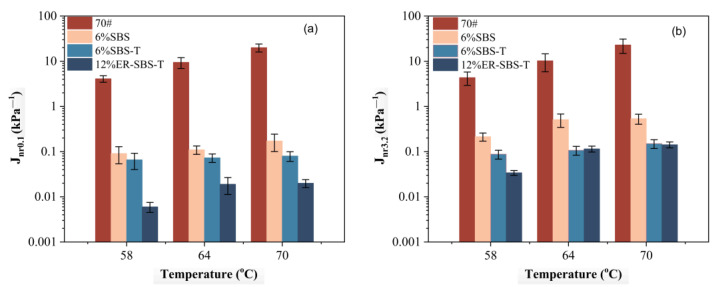
The average nonrecoverable creep under different stress levels and different temperatures. (**a**) 0.1 kPa, (**b**) 3.2 kPa.

**Figure 9:**
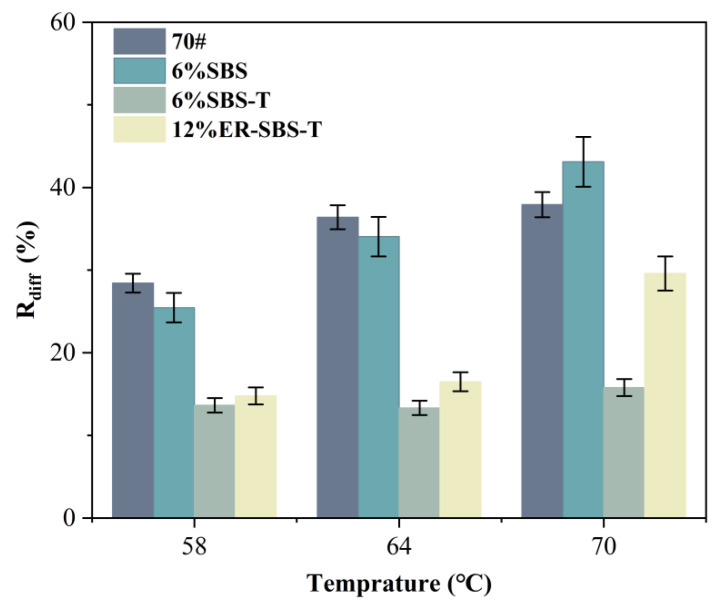
The results of R_diff_ at different temperatures.

**Figure 10:**
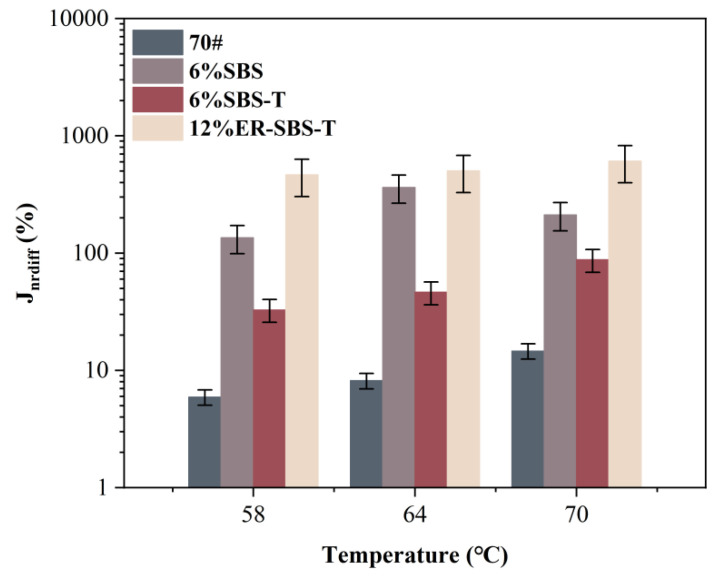
The results of J_nr-diff_ at different temperatures.

For the best high-temperature performance of modified asphalt binder, the appropriate amount of modifier should be selected based on the results of the above test. The improvement in the high-temperature performance of the asphalt binder is not obvious after the SBS modifier and SBS-T modifier are more than 6%. Therefore, 6% SBS-modified asphalt binder, 6% SBS-T-modified asphalt binder and 12% ER-SBS-T composite-modified asphalt binder were finally selected for the MSCR test. At the stress levels of 0.1 and 3.2 kPa, the average percent recovery R and average non-recoverable creep compliance J_nr_ of the modified asphalt binders at different temperatures are shown in Figure 7 and Figure 8. The percentage difference in average percent recovery R_diff_ and the percentage difference in non-recoverable creep compliance J_nr-diff_ were also calculated and plotted separately, as shown in Figure 9 and Figure 10. As the J_nr_ data range from 0.001 to 100, the log coordinates are used to directly compare different types of modified asphalt binders on the *y*-axis.

Figure 7 summarizes these conclusions; the size order is roughly ER-SBS-T composite-modified asphalt binder > SBS-modified asphalt binder > SBS-T-modified asphalt binder > 70# base asphalt binder from the R_0.1_. Based on the R_3.2_, the arrangement in order of size is roughly ER-SBS-T composite-modified asphalt binder > SBS-T-modified asphalt binder > SBS-modified asphalt > base asphalt. This indicates that the incorporation of modifiers increases the deformation recovery ability of asphalt binder. SBS-T-modified asphalt has a higher deformation recovery ability at lower stress levels than SBS-modified asphalt, while the opposite is true at higher stress levels. This shows that SBS-T has certain advantages over SBS in poorer road conditions. However, the ER-SBS-T composite-modified asphalt binder has the highest deformation recovery at high temperatures.

As the temperature increased, the average percent recovery of the asphalt binder decreased. This indicates that the increase in temperature changed the viscoelastic ratio of the asphalt binder, increasing the viscosity and decreasing the elasticity. The high-temperature deformation recovery ability of the asphalt binder is reduced. Different asphalt binders change with temperature, 70# base asphalt binder, and three modified asphalt binders change very little with temperature in terms of the R_0.1_. This indicates that the temperature sensitivity of some asphalt binders is modest at lower stress levels.

According to Figure 8, for the parameters Jnr_0.1_ and Jnr_3.2_, the size ranking is ER-SBS-T composite-modified asphalt binder < SBS-T-modified asphalt binder < SBS-modified asphalt binder < 70# base asphalt binder. The addition of the modifiers reduces the unrecoverable deformation of asphalt. The high-temperature performance of asphalt is also improved. Asphalt can resist permanent deformation. At different stress levels, the unrecoverable deformation of ER-SBS-T composite-modified asphalt binder is the lowest, indicating that the ER-SBS-T composite-modified asphalt binder has the strongest ability to resist permanent deformation. The SBS-T-modified asphalt binder is second, and the SBS-modified asphalt binder is the worst.

With increasing temperature, the average nonrecoverable creep of asphalt shows an upward trend, indicating that the unrecoverable deformation of asphalt increases with increasing temperature. Different asphalt binder specimens have different change rates with temperature. SBS-, SBS-T-, and ER-SBS-T-modified asphalt binders have little change in J_nr0.1_ with temperature, indicating that the temperature sensitivity of these three asphalt binders is not large at low stress levels. In view of Jnr_3.2_, the change range of the SBS-modified asphalt binder with temperature is very small, suggesting that the temperature sensitivity of SBS-modified asphalt binder is low at high load levels.

Figure 9 and Figure 10 present these discoveries. According to R_diff_, the grading is approximately 70# base asphalt binder > SBS-modified asphalt binder > ER-SBS-T composite-modified asphalt binder > SBS-T-modified asphalt binder. It shows that after the addition of the SBS-T modifier, the asphalt binder still has a strong recovery capacity at high stress levels, and the deformation resistance is higher than that of 70# base asphalt binder and SBS-modified asphalt binder. In the view of J_nr-diff_, the order is roughly 70# base asphalt binder < SBS-T-modified asphalt binder < SBS-modified asphalt binder < ER-SBS-T composite-modified asphalt binder. It shows that there is no significant difference in the mechanical response of 70# base asphalt binder in the linear viscoelastic intervals and the non-linear viscoelastic intervals. It is, therefore, not sensitive to changes in stress. The ER-SBS-T composite-modified asphalt binder is the largest, demonstrating that it is highly stress-sensitive. There is a big difference between the mechanical response of linear viscoelastic intervals and non-linear viscoelastic intervals. The traditional high-temperature evaluation method only evaluates the high-temperature performance of asphalt in linear viscoelastic intervals. The high-temperature performance evaluation of ER-SBS-T composite-modified asphalt binder is not comprehensive.

With increasing temperature, the R_diff_ and J_nr-diff_ of the various asphalt binders show an increasing trend, except for the J_nr-diff_ of the SBS-modified asphalt binder. The R_diff_ of ER-SBS-T composite-modified asphalt binder increases sharply at 70 °C compared to 64 °C, indicating that temperature has a great influence on the stress sensitivity of ER-SBS-T composite-modified asphalt binder. When the temperature reaches 70 °C, the viscoelastic properties of the asphalt change significantly, and the non-linear viscoelasticity becomes more significant at the same high stress. J_nr-diff_ at 70 °C decreased when compared with 64 °C, which may be due to the poor deformation resistance of SBS-modified asphalt binder, and the changing trend was not obvious.

### 3.4. Influence of Modifier Dosage on MSCR-Related Parameters

**Figure 11:**
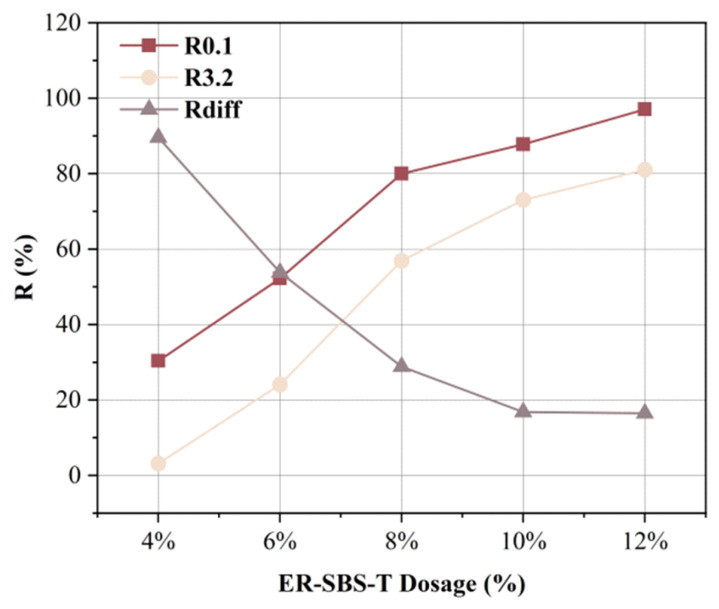
The results of R at different dosages.

**Figure 12:**
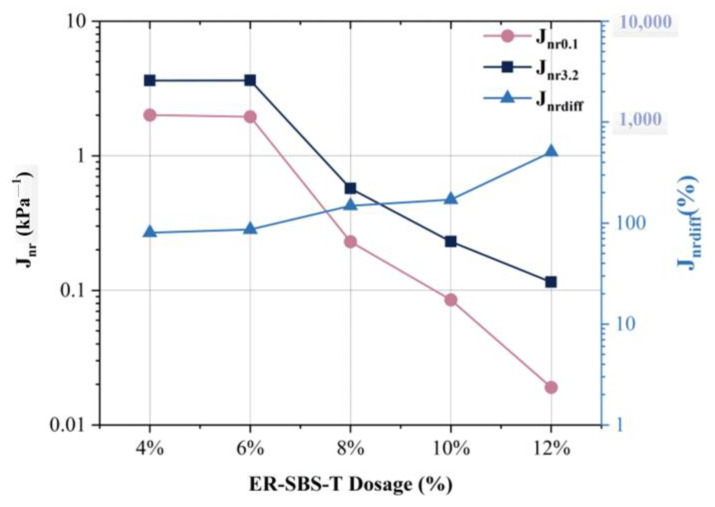
The results of J_nr_ at different dosages.

The temperature was 64 °C, and the dosages of ER-SBS-T composite modifier were 4%, 6%, 8%, 10% and 12%. The average percent recovery R_0.1_, R_3.2_, and average non-recoverable creep Jnr_0.1_, Jnr_3.2_ of the modified asphalt were measured, and R_diff_ and Jnr-diff were calculated. The broken line plots are described in Figure 11 and Figure 12. Figure 11 summarizes that R_0.1_ and R_3.2_ increased with the increase in ER-SBS-T composite modifier content, indicating that the recoverability of ER-SBS-T composite-modified asphalt binder is gradually increased. As R_0.1_ gradually approaches 100% with the increase in dosage, the growth rate slows down, resulting in a greater growth rate of R_3.2_ than R_0.1_. R_diff_ gradually decreases with the increase in dosage, indicating that the average percentage recovery of asphalt under high stress and low stress gradually converges with the increase in content.

According to Figure 12, J_nr0.1_ and J_nr3.2_ gradually decreased as the amount of ER-SBS-T composite modifier increased, suggesting that the permanent deformation resistance of the modified asphalt gradually increased with the increase in the content, and the stress sensitivity parameters J_nr-diff_ gradually increased with the increase in content. This indicates that the difference in creep of asphalt under high stress and low stress increases with the increase in dosage. Jnr3.2 decreases more slowly than Jnr0.1 with the increase in dosage, resulting in the gradual increase in the Jnr-diff with the change in dosage, which indicates that the improvement of high-temperature properties of asphalt under low stress and high stress is different with the addition of the ER-SBS-T modifier. The modified asphalt is mainly used to improve the high-temperature performance of asphalt in the linear viscoelastic range.

### 3.5. Pavement Performance Grading Based on the MSCR Test

According to the method provided by the AASHTO MP 19-10 [35] specification and the corresponding pavement performance grading by J_nr3.2_, the rutting performance is divided into extremely heavy traffic (E), very heavy traffic (V), heavy traffic (H), and standard traffic (S). The specific classification requirements are proposed in Table 4. According to the requirements in Table 4, The pavement performance grading is shown in Table 5.

**Figure 13:**
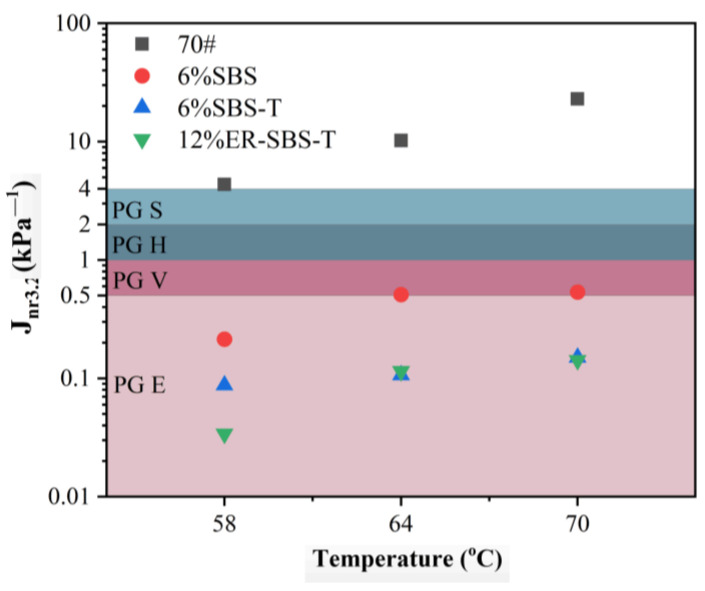
Pavement performance grading of the different modified asphalt binders.

**Figure 14:**
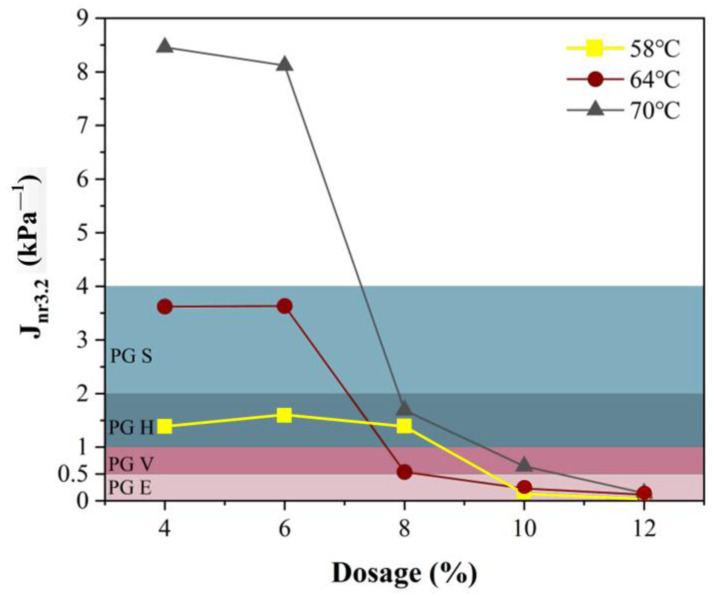
Pavement performance grading of the ER-SBS-T composite-modified asphalt binder.

The pavement performance grading of 70# base asphalt binder, 6% SBS-modified asphalt binder, 6% SBS-T-modified asphalt binder, and 12% ER-SBS-T composite-modified asphalt binder at 58 °C, 64 °C, 70 °C was performed, according to the results in Table 5. The results are indicated in Figure 13. The pavement performance gradings of ER-SBS-T composite-modified asphalt binder at temperatures of 58 °C, 64 °C, and 70 °C are exhibited in Figure 14. Figure 13 shows the pavement performance grading of different modified asphalt binders. At 58 °C, the 70# base asphalt binder cannot meet the requirements of standard traffic. The other modified asphalt binder is graded as PG 58 E, which meets the requirements of extremely heavy traffic. At 64 °C, 70# base asphalt cannot meet the requirements of standard traffic, and the grade of SBS-modified asphalt binder is PG 64 V, which meets the requirements of very heavy traffic. The classification of SBS-T-modified asphalt binder and ER-SBS-T composite-modified asphalt binder is PG 64 E, which meets the requirements of extremely heavy traffic. At 70 °C, 70# base asphalt cannot meet the requirements of standard traffic, and the classification of SBS-modified asphalt binder is PG 70 V, while the classifications of SBS-T-modified asphalt binder and ER-SBS-T composite-modified asphalt binder are PG 70 V.

Figure 14 summarizes the pavement performance grading of ER-SBS-T composite-modified asphalt binder. The difference in the high-temperature characteristics becomes increasingly apparent as the temperature rises. Therefore, 70 °C was chosen as the reference temperature and the J_nr3.2_ was used as the grading index. At 70 °C, the 4% and 6% ER-SBS-T composite-modified asphalt binders cannot meet the requirements of standard traffic. The 8% ER-SBS-T composite-modified asphalt binder was classified as PG 70 H, meeting the requirements of heavy traffic. The 10% ER-SBS-T composite-modified asphalt binder has been classified as PG 70 V, which meets the requirements of very heavy traffic. The 12% ER-SBS-T composite-modified asphalt binder was classified as PG 70 E, which meets the requirements of extremely heavy traffic. This shows that the PG based on MSCR can significantly discriminate the ER-SBS-T composite-modified asphalt binder at high loads and high temperatures.

### 3.6. Tests Results for ZSV Test

**Figure 15:**
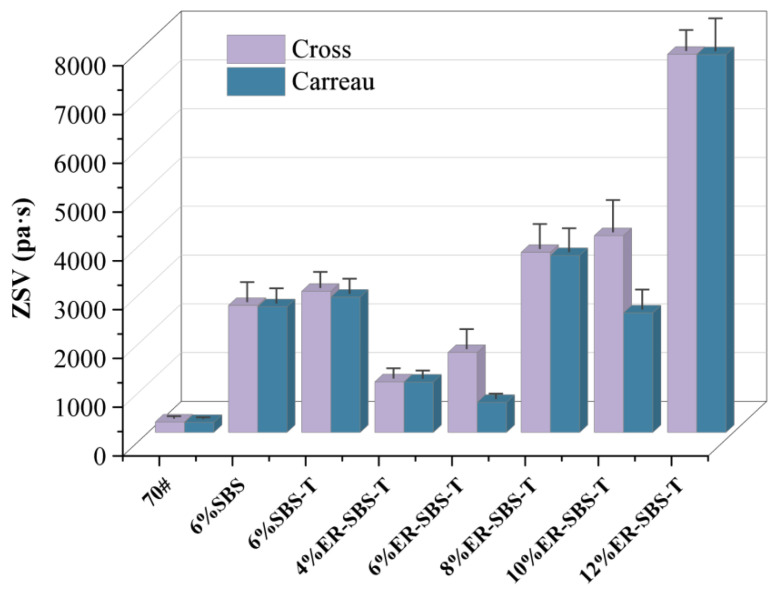
Zero-shear viscosity outcomes obtained from two rheological models.

Based on the formula of the Carreau and Cross rheological models, the OriginPro software (Learning Edition) was used for η−ω curve fitting. The zero-shear viscosity, model difference, and relative coefficient R^2^ of the 70# base and modified asphalt binders of each batch can be obtained, as shown in Table 6. The zero-shear viscosity data from Table 6 were plotted, as shown in Figure 15. The relative coefficients R^2^ of various modified asphalt binder after fitting of Cross model and Carreau models were greater than 0.95, indicating that these two models have a high fit to the modified asphalt binder, and the relative coefficient R^2^ of the 70# base asphalt binder is 0.94 and 0.92. Compared to this modified asphalt binder, the relative coefficient is poor, which may be caused by the softening and deformation of the base asphalt on the 25 mm parallel plate fixture, which results in deviation in the measured data. Therefore, there is a large error in the fit of the 70# asphalt binder with these two models.

The fitting results of the Cross model are all greater than those of the Carreau model. The model difference between base asphalt, SBS-modified asphalt binder, and SBS-T-modified asphalt binder is small, while the model difference in the ER-SBS-T composite-modified asphalt binder is large. The model difference of 6% dosage is 62.33%, and the model difference of 10% content is 39.23%, indicating that the fitting results of the two models for ER-SBS-T composite-modified asphalt binder are quite different. From the point of view of the ER-SBS-T composite-modified asphalt binder with different dosages, the fitting results of the Cross model gradually increase with the increase in the dosage, and when the dosage is greater than 10%, the performance jumps significantly, which is consistent with the above test conclusion, while the Carreau model has no significant rule. This indicates that the Cross model is reasonable for evaluating the zero-shear viscosity of ER-SBS-T composite-modified asphalt binder.

## 4. Conclusions

In this investigation, by analyzing the asphalt penetration, softening point, average percent recovery, average nonrecoverable creep, stress sensitivity parameters, pavement performance grading based on MSCR, and the zero-shear viscosity parameters, the following conclusions can be drawn:

- Compared to the SBS-modified asphalt binder, the high-temperature performance of the SBS-T-modified asphalt binder is slightly better, and the high-temperature performance of the two modified asphalt binders stops improving after the content exceeds 6%, while the addition of the ER-SBS-T composite modifier can continue to enhance the high-temperature performance of the asphalt binder, and the high-temperature performance keeps improving after the content exceeds 6%. When the dosage is greater than 10%, the high-temperature performance jumps significantly, and it is better than the 6% SBS-modified asphalt binder, and the 6% SBS-T-modified asphalt binder. Therefore, in practice, a 6% dosage of the SBS- and SBS-T-modified asphalt binder can be chosen. For the ER-SBS-T composite-modified asphalt binder, a dosage of 10% or more can be chosen.
- The high-temperature performance of ER-SBS-T composite-modified asphalt at 58 °C, 64 °C, and 70 °C are all excellent. The temperature sensitivity of SBS-T-modified asphalt, SBS-modified asphalt, and ER-SBS-T composite-modified asphalt is low, and the high-temperature performance and stress sensitivity do not change significantly with the change in test temperature.
- The improvement in the high-temperature performance of asphalt binder at low and high stress levels is different with the addition of the ER-SBS-T composite modifier. This is due to the fact that this modifier improves the high-temperature performance of asphalt in the linear viscoelastic interval.
- For the penetration, is difficult to reflect the high-temperature performance of the SBS-T-modified asphalt. The average percentage recovery, average non-recoverable creep, and zero-shear viscosity fitted by the Cross model can reflect the high-temperature performance of the modified asphalt binder, and the 70 °C pavement performance grading can effectively distinguish the high-temperature performance of these three modified asphalt binders.
- Based on the PG system of MSCR, 70 °C can be used as the reference temperature for performance classification of the ER-SBS-T composite-modified asphalt binder when the content is less than 12%. The reference temperature for performance grading of the ER-SBS-T composite-modified asphalt binder at higher blends needs to be further investigated.

## Figures and Tables

**Table 1:** Properties of 70# base asphalt binder.

Technical Parameter	Units	Values	Method
Penetration (25 °C)	0.1 mm	72.4	T0604-2011
Ductility (15 °C, 5 cm/min)	cm	114	T0605-2011
Softening point	°C	47.7	T0606-2011

**Table 2:** Technical information of modifiers.

Modifiers	Parameters	Units	Values
SBS	S/B ratio	—	28/72
	Tensile strength	MPa	34.1
	Elongation	%	761
	Oil content	%	0.7
	Melt flow rate	g/10 min	0.82
SBS-T	Individual particle quality	g	0.21
	Ash content	%	0.48
	Melting index	g/10 min	2.39
ER-SBS-T	Individual particle quality	g	<0.001
	Ash content	%	1.4
	Melting index	g/10 min	13.8
	Density	g/cm^3^	0.975

**Table 3:** 60 °C kinematic viscosity of modified asphalt binder under different dosage of modifiers.

Parameters	Modifiers	Dosage				
		4%	6%	8%	10%	12%
60 °C kinematic viscosity (Pa∙s)	SBS	3600	12,800	18,900	—	—
	SBS-T	5900	16,700	22,000	—	—
	ER-SBS-T	2800	6000	19,000	58,000	148,900
Rate of change in the viscosity (%)	SBS	—	258	47	—	—
	SBS-T	—	183	34	—	—
	ER-SBS-T	—	114	223	203	156

**Table 4:** AASHTO MP 19-10 pavement performance grading method.

Traffic Volume Classification	Test Temperature			Jnr_3.2_/kPa^−1^	J_nr-diff_/%
	58 °C	64 °C	70 °C		
Extremely heavy traffic (more than 30 million axles)	PG58E	PG64E	PG70E	≤0.5	≤75
Very heavy traffic (more than 30 million axles)	PG58V	PG64V	PG70V	≤1.0	≤75
heavy traffic (10 million to 30 million axles)	PG58H	PG64H	PG70H	≤2.0	≤75
Standard traffic (Less than 10 million axles)	PG58S	PG64S	PG70S	≤4.0	≤75

**Table 5:** Pavement performance grading of modified asphalt binders.

Bitumin	Jnr_3.2_/kPa^−1^			Pavement Performance Grading		
	58 °C	64 °C	70 °C	58 °C	64 °C	70 °C
70#	4.34	10.22	22.94	—	—	—
6% SBS	0.21	0.511	0.54	PG58E	PG64V	PG70V
6% SBS-T	0.09	0.11	0.15	PG58E	PG64E	PG70E
4% ER-SBS-T	1.39	3.62	8.46	PG58H	PG64S	—
6% ER-SBS-T	1.60	3.63	8.11	PG58H	PG64S	—
8% ER-SBS-T	1.4	0.57	1.69	PG58H	PG64V	PG70H
10% ER-SBS-T	0.14	0.23	0.64	PG58E	PG64E	PG70V
12% ER-SBS-T	0.03	0.12	0.14	PG58E	PG64E	PG70E

**Table 6:** Zero-shear viscosity parameters fitted by two models.

Bitumin	Zero-Shear Viscosity (Pa·s)			Relative Coefficient R^2^	
	Cross Model	Carreau Model	Model Difference (%)	Cross Model	Carreau Model
70#	216.05	206.66	4.34	0.94	0.92
6% SBS	2604.78	2651.30	1.04	0.98	0.99
6% SBS-T	2894.25	2998.60	4.11	0.99	0.99
4% ER-SBS-T	1038.58	1034.09	0.43	0.98	0.98
6% ER-SBS-T	1641.04	618.16	62.33	0.99	0.99
8% ER-SBS-T	3693.81	3628.68	1.76	0.96	0.97
10% ER-SBS-T	4035.56	2452.29	39.23	0.99	0.99
12% ER-SBS-T	7753.25	7766.17	0.09	0.96	0.99

## Data Availability

All data, models, and codes generated or used during the study appear in the submitted article.
